# Supplementary material for: Physical origin of giant excitonic and magneto-optical responses in two-dimensional ferromagnetic insulators
Source: Nat Commun. 2019 May 30;10:2371. doi: 10.1038/s41467-019-10325-7 (PMC6542836; doi:10.1038/s41467-019-10325-7)
Supplement: Supplementary file 1 — Supplementary Information [file 41467_2019_10325_MOESM1_ESM.pdf]

Supplementary Information for "Physical origin of giant excitonic and magneto-optical responses in two-dimensional ferromagnetic insulators"

Wu et al.

A. G_0W_0 calculations using LSDA+U as the starting point

In our G_0W_0 calculations, we will treat on the same footing the Hubbard potential (V_{Hub}) and the LSDA exchange-correlation potential $V_{\mathrm{xc}}^{\mathrm{LSDA}}$. That is, the self-energy correction is given by [1-4],

$$\Delta\Sigma = \Sigma - V_{\mathrm{xc}} - V_{\mathrm{Hub}}, \tag{1}$$

where Σ is the conventional self-energy operator in the GW approximation. In this way, the Hubbard potential, together with the LSDA exchange-correlation potential, have been subtracted from the quasiparticle energies corrections to avoid the double-counting issue. As shown in Supplementary Figure 1 and 2, the GW (at the G_0W_0 level) band structure and GW-BSE absorption spectrum exhibit only a weak dependence on U and J in a physically reasonable range of values [2-4].

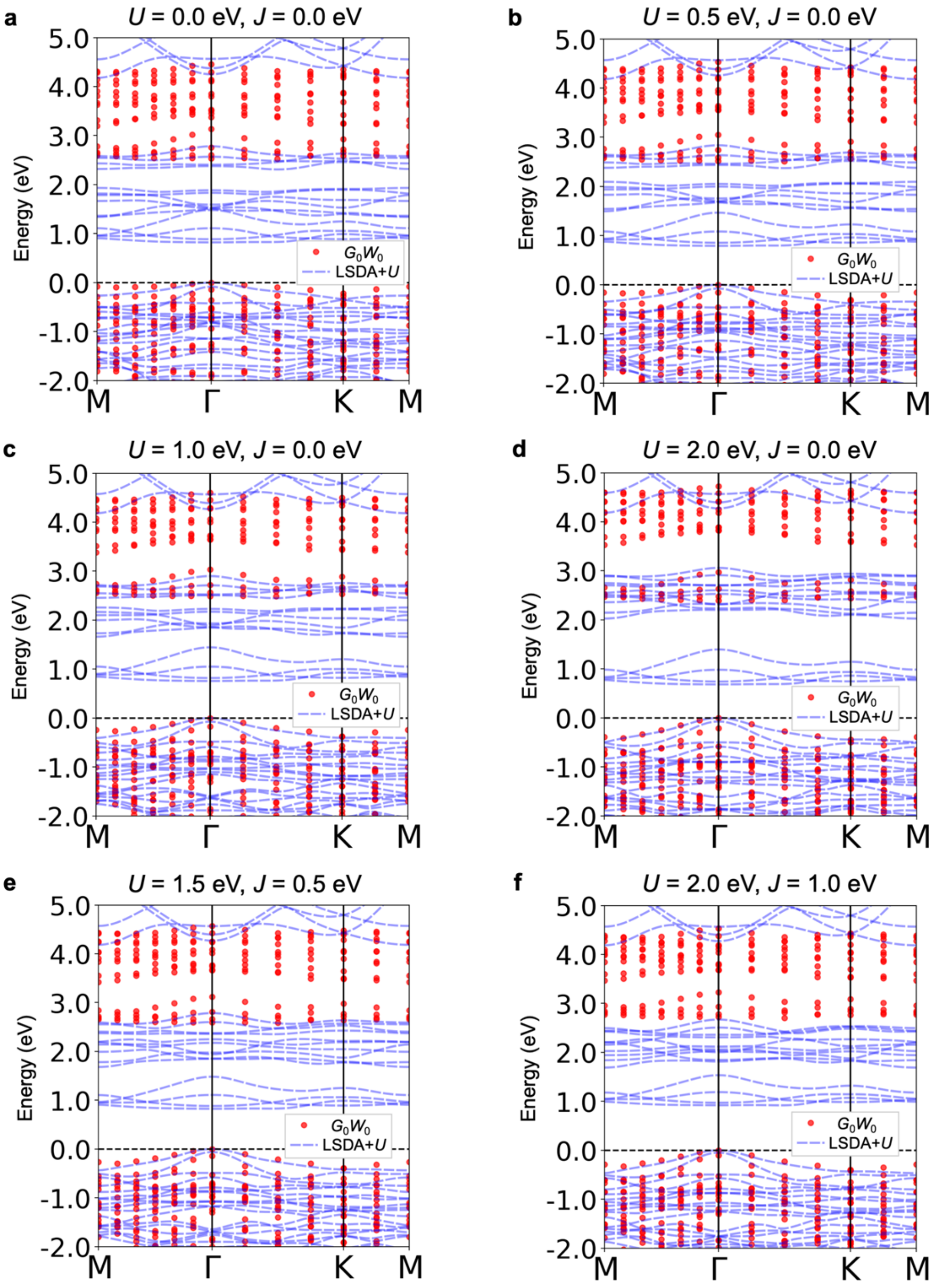

Supplementary Figure 1 | Dependence of the G_0W_0 band structure on parameters of U and J within the LSDA+U scheme. We consider the following sets of parameters: (**a**) $U = 0$ eV, $J = 0$ eV (**b**) $U = 0.5$ eV, $J = 0.0$ eV, (**c**) $U = 1.0$ eV, $J = 0.0$ eV, (**d**) $U = 2.0$ eV, $J = 0.0$ eV, (**e**) $U = 1.5$ eV, $J = 0.5$ eV, and (**f**) $U = 2.0$ eV, $J = 1.0$ eV.

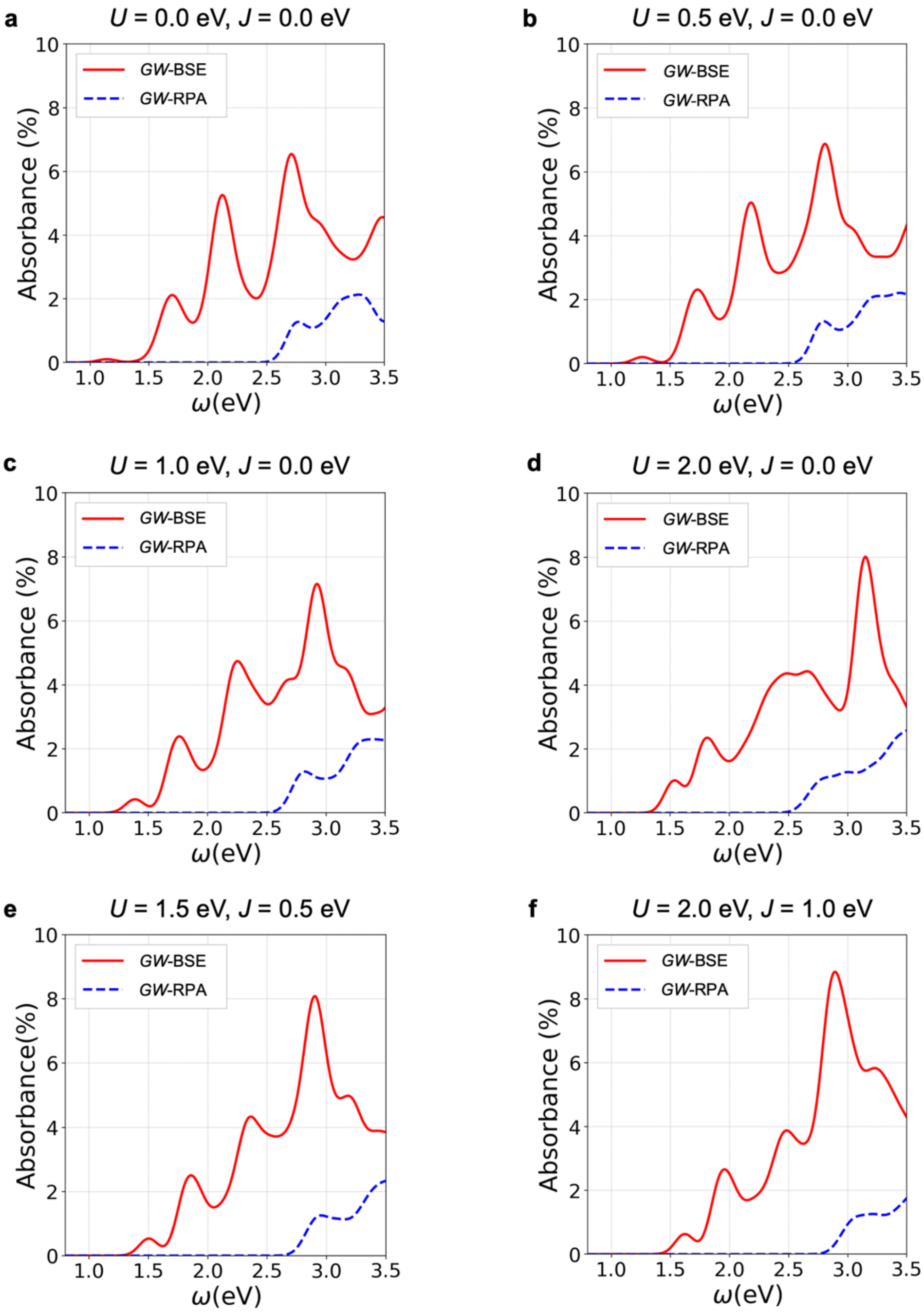

Supplementary Figure 2 | Dependence of the linear absorption spectrum at normal incidence on parameters of U and J within the LSDA+U scheme, with (GW-BSE, solid red line) and without (GW-RPA, dashed blue line) electron-hole interaction. We consider the following sets of parameters: (**a**) $U = 0$ eV, $J = 0$ eV (**b**) $U = 0.5$ eV, $J = 0.0$ eV, (**c**) $U = 1.0$ eV, $J = 0.0$ eV, (**d**) $U = 2.0$ eV, $J = 0.0$ eV, (**e**) $U = 1.5$ eV, $J = 0.5$ eV, and (**f**) $U = 2.0$ eV, $J = 1.0$ eV.

B. Density of states (DOS) of ferromagnetic monolayer CrI_3

The partial DOS (PDOS) based on the G_0W_0 band structure are shown in Supplementary Figure 3. We use spinor Wannier functions to interpolate the band structure as implemented in Wanner90 [5]. According to the calculated PDOS in Supplementary Figure 3, the first manifold of quasi-particle conduction bands (2.5 eV ~ 3.1 eV) consist of nearly equal contributions from Cr major-spin 3*d* and I major-spin 5*p* states, while the upper conduction bands (> 3.1 eV) are dominated by minor-spin Cr 3*d* states; and the top of valence bands host a decent amount of Cr major-spin 3*d* states, as well as the dominant I major-spin 5*p* states. In this way, the carrier energy distribution and the spatial localization of the dark state (Fig. 3a&e in the main text) origin from intra-atomic *d*-*d* transitions with spin flip. The contributions from occupied I 5*s* orbitals are around -12 eV below the highest occupied state, and those from occupied Cr 3s, 3*p* and 4*s* orbitals are below -40 eV.

It is obvious that, compared to LSDA, LSDA+*U* pushes the major-spin *d* states downwards in energy and significantly change the hybridization between the major-spin *d* states and the valence *p* states. However, LSDA+*U* has relatively small effects on the conduction states.

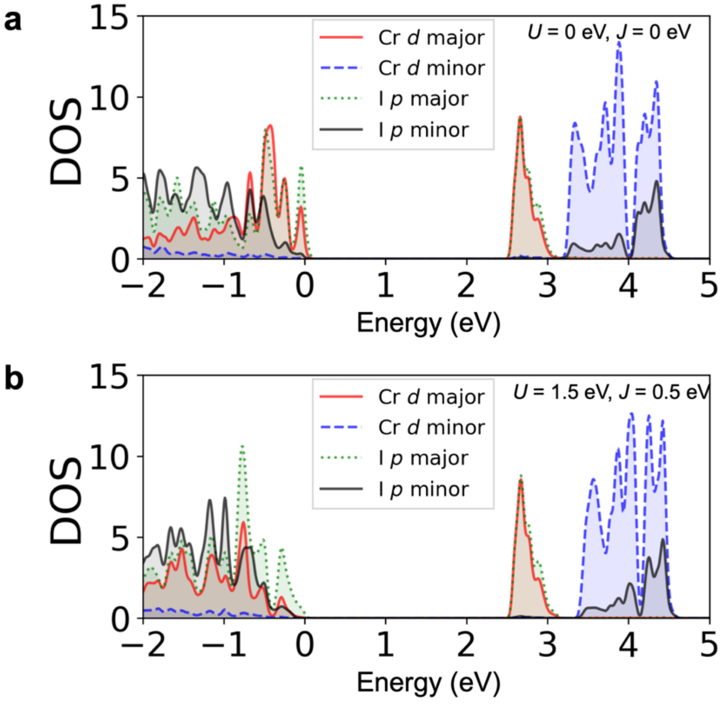

Supplementary Figure 3 | Partial DOS of a ferromagnetic monolayer CrI_3 for (**a**) $U = 0$ eV, $J = 0$ eV and (**b**) $U = 1.5$ eV, $J = 0.5$ eV. The DOS is decomposed into contributions from Cr major-spin 3*d* orbitals

(solid red curve), Cr minor-spin 3*d* orbitals (dashed blue curve), I major-spin 5*p* orbitals (green dotted curve) and I minor-spin 5*p* orbitals (black solid curve). The energy of the VBM is set to zero.

C. Numerical evaluation of exciton radius

We use the exciton wave functions shown in Fig. 2 and Fig. 3 in the main text to evaluate the arithmetic mean radius $\langle|\mathbf{r}|\rangle$ and the root mean square radius $\sqrt{\langle r^2\rangle}$ as shown in Supplementary Table 1. Our first-principles results agree with the intuition that a large binding energy indicates a small exciton radius, as inspired by a 2D hydrogenic model.

Exciton states	A	B^+	B^-	C
$\boldsymbol{\Omega_S}$ (eV)	1.50	1.82	1.92	2.31
E_b (eV)	1.09	0.77	0.67	0.28
$\langle|\mathbf{r}|\rangle$ (Å)	2.33	3.55	5.36	6.99
$\sqrt{\langle \boldsymbol{r^2}\rangle}$ (Å)	3.06	4.37	6.70	7.93

Supplementary Table 1 | The arithmetic mean radius $\langle|\mathbf{r}|\rangle$ and root mean square radius $\sqrt{\langle r^2\rangle}$ of selected bright exciton states. The excitation energy Ω_S and binding energy E_b are also included for reference.

D. Spin-decomposed exciton probability amplitudes

Since the two-component spinor wave functions are used in our calculations, it is possible for us to choose certain spinor components of wave functions and calculate the spin configuration of constituent carriers for each exciton state. To be specific, an exciton wave function in the spinor formalism can be written as,

$$\boldsymbol{\Psi}(\mathbf{r}_e,\mathbf{r}_h)=\sum_{v c \mathbf{k}} A^S_{vc\mathbf{k}}\boldsymbol{\psi}_{c\mathbf{k}}(\mathbf{r}_e)\boldsymbol{\psi}^*_{v\mathbf{k}}(\mathbf{r}_h):=\sum_{vc\mathbf{k}} A^S_{vc\mathbf{k}}|c\rangle\langle v| \\ =\sum_{vc\mathbf{k}} A^S_{vc\mathbf{k}}\begin{pmatrix}|c\uparrow\rangle\langle v\uparrow| & |c\uparrow\rangle\langle v\downarrow| \\ |c\downarrow\rangle\langle v\uparrow| & |c\downarrow\rangle\langle v\downarrow|\end{pmatrix}. \tag{2}$$

The fractions for each spin configuration are listed in Supplementary Table 2.

$\left|\sum_{vc\mathbf{k}} A^S_{vc\mathbf{k}}|c\rangle\langle v|\right|^2$	$|c\uparrow\rangle\langle v\uparrow|$	$|c\uparrow\rangle\langle v\downarrow|$	$|c\downarrow\rangle\langle v\uparrow|$	$|c\downarrow\rangle\langle v\downarrow|$
Ω_S=0.89 eV	0.4%	0.0%	99.5%	0.1%
Ω_S=1.50 eV	98.7%	0.1%	1.2%	0.0%

Ω_S=1.82 eV	98.6%	0.3%	1.1%	0.0%
Ω_S=1.92 eV	97.8%	1.2%	1.0%	0.0%
Ω_S=2.31 eV	96.4%	1.1%	2.5%	0.0%

Supplementary Table 2 | Decomposition of carrier spin configurations for selected exciton states.

E. Computational details for Si and SiO_2

The *GW* (at G_0W_0 level) and *GW*-BSE calculations of bulk Si are performed with the BerkeleyGW code [6]. The experimental lattice constant of a = 5.43 Å at 300 K is adopted in the calculations. The resulting frequency-dependent dielectric function of Si is shown in Supplementary Figure 4. Note that $\varepsilon_{xy} = 0$ for bulk silicon and we could use a scalar dielectric function $\varepsilon = \varepsilon_{xx} = \varepsilon_{yy} = \varepsilon_{zz}$ for cubic crystals such as Si and SiO_2.

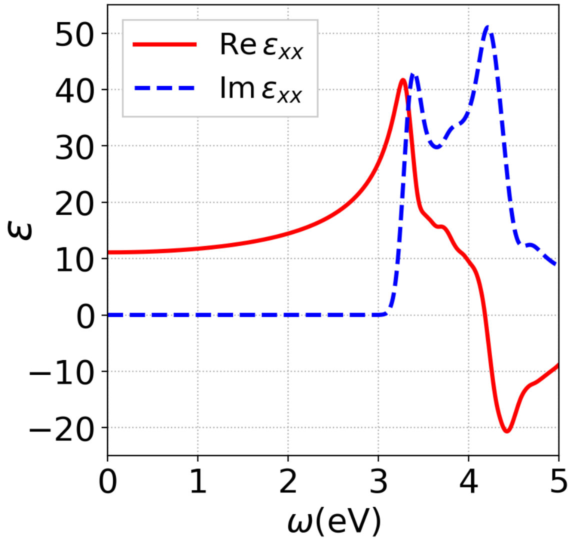

Supplementary Figure 4 | Dielectric function ε_{xx} of bulk Si from *GW*-BSE calculations. An 80 meV Gaussian broadening is employed.

The reflective index for bulk Si is calculated as,

$$n(\omega) = \sqrt{\varepsilon(\omega)}, \quad (3)$$

where the static value $\mathrm{Re}[\varepsilon(\omega \to 0)] = 11.1$ is in excellent agreement with experimental value of 11.68 or 11.4 [7, 8].

For bulk SiO_2, because the band gap (8.9 eV) is much larger than the energy range of interest in this problem (< 3.5 eV), we will only consider a static reflective index (with experimental value) with a very small imaginary part (~0.01i) to account for other dissipation channels [9],

$$n^2 = \varepsilon(\omega \to 0) = 3.9. \tag{4}$$

F. Normal Modes

In the P-MOKE configuration with at least C_3 rotational symmetry along the magnetization direction ($\mathbf{B} = B\hat{\mathbf{e}}_z$), the dielectric tensor takes the following form,

$$\boldsymbol{\varepsilon}(\omega, \mathbf{B}) = \begin{pmatrix} \varepsilon_{xx}(\omega, \mathbf{B}) & \varepsilon_{xy}(\omega, \mathbf{B}) & 0 \\ -\varepsilon_{xy}(\omega, \mathbf{B}) & \varepsilon_{xx}(\omega, \mathbf{B}) & 0 \\ 0 & 0 & \varepsilon_{zz}(\omega, \mathbf{B}) \end{pmatrix}. \tag{5}$$

The Fresnel equation is given by,

$$[n^2\mathbb{I} - \boldsymbol{\varepsilon} - \mathbf{n}:\mathbf{n}] \cdot \mathbf{E} = 0, \tag{6}$$

where $\mathbf{n}$ is the complex refractory index,

$$\mathbf{n} = \frac{c\mathbf{k}}{\omega}. \tag{7}$$

After solving the Fresnel equations with the dielectric function in Supplementary Eq. 5 with $\mathbf{k} \parallel \hat{\mathbf{e}}_z$, we get the normal modes as the σ^+ and σ^- circularly polarized plane waves, with distinct refractive indices,

$$n_\pm^2(\omega, B\hat{\mathbf{e}}_z) = \varepsilon_{xx}(\omega, B\hat{\mathbf{e}}_z) \pm i\varepsilon_{xy}(\omega, B\hat{\mathbf{e}}_z), \tag{8}$$

where the $+(-)$ in $n_\pm$ denotes the circularly polarized light with the complex electric field amplitude along the direction of the spherical basis:

$$\hat{\mathbf{e}}_\pm = \frac{\mp}{\sqrt{2}}(\hat{\mathbf{e}}_x \pm i\hat{\mathbf{e}}_y). \tag{9}$$

G. Kerr signals and Faraday signals

Following the convention in previous works [10], we get the ratio of complex amplitude of $\sigma^\pm$ circularly polarized reflection light through the ratio of the corresponding complex reflectivity $\tilde{r}^{(\pm)}$ as,

$$\frac{\tilde{E}^{(-)}{}_r}{\tilde{E}^{(+)}{}_r} = \frac{\tilde{r}^{(-)}}{\tilde{r}^{(+)}}. \tag{10}$$

Suppose that an incident linearly polarized light is along the x-axis, the relative complex amplitudes of the reflection light $\tilde{\mathbf{E}}_r$ (at fixed z and t) along x- and y-axis are then given by,

$$\frac{\tilde{E}_{rx}}{\tilde{E}_{ry}} = \frac{1 + \tilde{r}^{(-)}/\tilde{r}^{(+)}}{i(1 - \tilde{r}^{(-)}/\tilde{r}^{(+)})}, \quad (11)$$

which defines an ellipse oriented slightly away from the *x*-axis as shown in Supplementary Figure 5b.

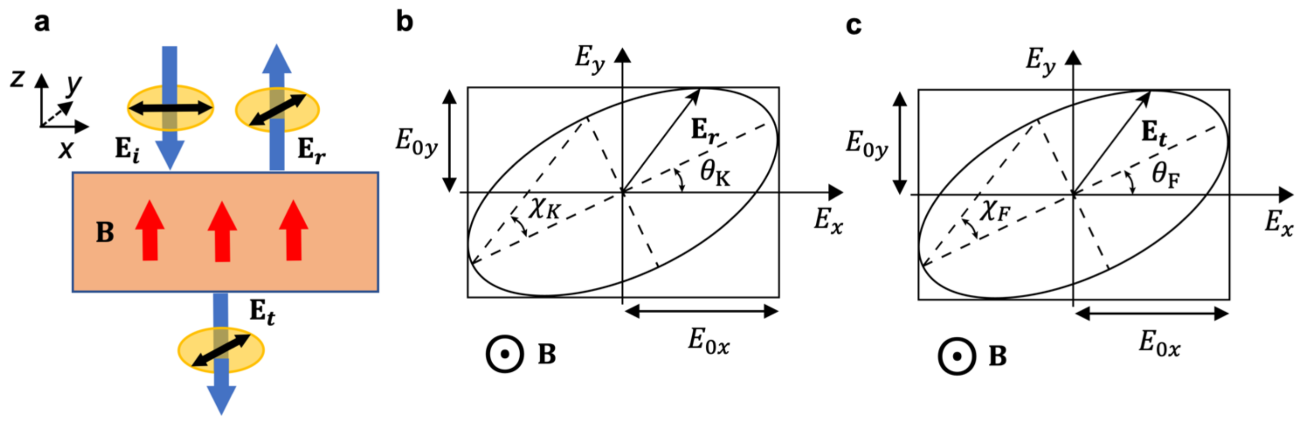

Supplementary Figure 5 | (a) Configuration of the polar MO effects. The red arrows denote the magnetization of the sample, which is pointing along the +*z* direction. (**b**) The polarization plane of the reflection light. The polarization ellipse is oriented at a Kerr angle θ_K with respect to the *x*-axis. The Kerr ellipticity is defined through the ellipticity angle χ_K as shown. (**c**) The polarization plane of the transmission light. The polarization ellipse is oriented at a Faraday angle θ_F with respect to the *x*-axis. The ellipticity is defined through the Faraday ellipticity angle χ_F.

We can calculate the Kerr angle θ_K and Kerr ellipticity χ_K as [11],

$$\tan 2\theta_K = \frac{2E_{0x}E_{0y}\cos\delta}{E_{0x}^2 - E_{0y}^2}, -\frac{\pi}{2} < \theta_K \leq \frac{\pi}{2} \quad (12)$$

and

$$\sin 2\chi_K = \frac{2E_{0x}E_{0y}\sin\delta}{E_{0x}^2 + E_{0y}^2}, -\frac{\pi}{4} < \chi_K \leq \frac{\pi}{4} \quad (13)$$

where $\delta = angle(\tilde{E}_{0y}/\tilde{E}_{0x})$, $E_{0x} = |\tilde{E}_{0x}|$ and $E_{0y} = |\tilde{E}_{0y}|$, and *angle(Z)* is a function that returns the phase angle of a complex number *Z*. A sign convention enters the expression of Kerr angle θ_K: θ_K is chosen to be positive if the rotation vector of the polarization plane is parallel to the magnetization vector.

The Faraday angle θ_F and Faraday ellipticity χ_F are defined in a similar way for the transmission light, as shown in Supplementary Figure 5c.

H. Multi-interface P-MOKE Setup

Here we model the multi-interface P-MOKE setup in a systematic way [12, 13]. The goal is to calculate the complex reflection coefficients for $\sigma^{\pm}$ circularly polarized light, $\tilde{r}^{(\pm)} = \tilde{E}_r^{(\pm)} / \tilde{E}_i^{(\pm)}$ at the interface. The resulting complex reflection coefficients (at the topmost interface) for the three-interface model (shown in Fig. 4a in the main text) are given by,

$$\tilde{r}^{(\pm)} = \frac{e^{2i\delta_1^{(\pm)}}\left(\tilde{r}_{12}^{(\pm)} + e^{2i\delta_2}\tilde{r}_{23}\right) + e^{2i\delta_2}\tilde{r}_{01}^{(\pm)}\tilde{r}_{12}^{(\pm)}\tilde{r}_{23} + \tilde{r}_{01}^{(\pm)}}{1 + e^{2i(\delta_1^{(\pm)}+\delta_2)}\tilde{r}_{01}^{(\pm)}\tilde{r}_{23} + e^{2i\delta_1^{(\pm)}}\tilde{r}_{01}^{(\pm)}\tilde{r}_{12}^{(\pm)} + e^{2i\delta_2}\tilde{r}_{12}^{(\pm)}\tilde{r}_{23}}. \quad (14)$$

where the one-interface complex reflection coefficient between the i-th layer and the j-th layer is defined by $\tilde{r}_{ij} = (\tilde{n}_i - \tilde{n}_j)/(\tilde{n}_i + \tilde{n}_j)$, and the light path within the i-th layer is defined as $\delta_i = \omega\tilde{n}_i d_i/c$. The two-interface model can be achieved by taking $e^{i\delta_2} \to 0$. The one-interface model can be achieved by taking $e^{i\delta_1} \to 0$ and $e^{i\delta_2} \to 0$.

I. Effects of substrate thickness on MO signals

Here we investigate the influence of substrate thickness on MO signals. As described in the main text, we construct a three-interface P-MOKE setup with the order of vacuum-CrI_3-SiO_2-Si. We vary the thickness of the SiO_2 layer ($d_{\mathrm{SiO_2}}$) and assume a semi-infinitely thick Si layer. We find that the interference of light reflected on each interface will be very sensitive to $d_{\mathrm{SiO_2}}$. To be explicit, the amplitudes of the MOKE signals present a sensitive dependence on the substrate (Supplementary Figure 6), and it will be strongly modulated in the energy range of interest (1.0 ~ 3.5 eV) with increasing thickness of $d_{\mathrm{SiO_2}}$, as shown in Supplementary Figure 6.

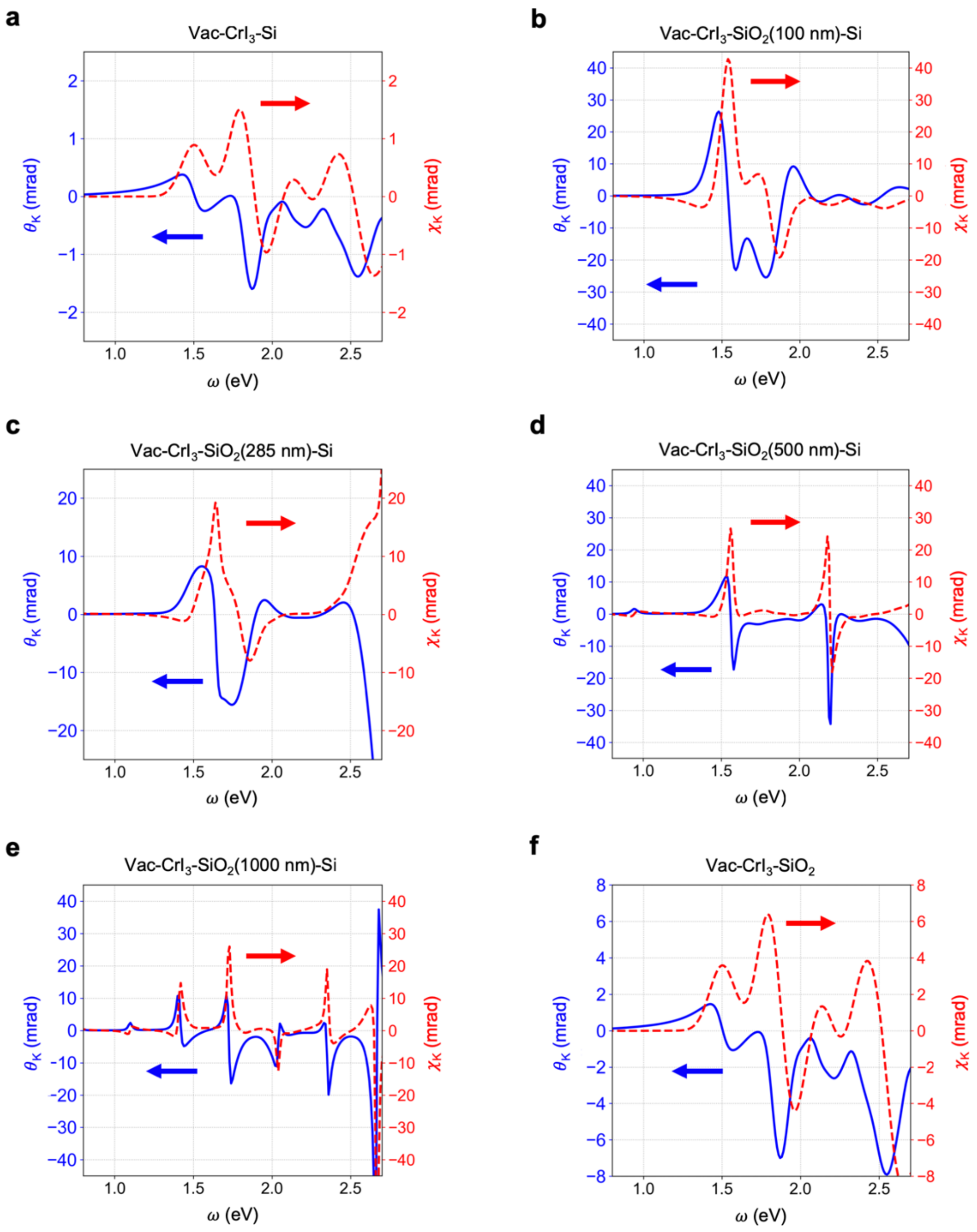

Supplementary Figure 6 | Kerr angles θ_K (left, solid blue curve) and Kerr ellipticity χ_K (right, dashed red curve) for different P-MOKE setups with (**a**) vacuum-CrI_3-Si, (**b**) vacuum-CrI_3-SiO_2(100 nm)-Si, (**c**) vacuum-CrI_3-SiO_2(285 nm)-Si, (**d**) vacuum-CrI_3-SiO_2(500 nm)-Si, (**e**) vacuum-CrI_3-SiO_2(1000 nm)-Si and (**f**) vacuum-CrI_3-SiO_2 interfaces. All the CrI_3 layers in (**a-f**) refer to ferromagnetic monolayer CrI_3.

J. In-plane ferromagnetic monolayer CrI_3

Our LSDA+U and G_0W_0 calculations have reproduced the reported magneto band structure effect where relevant bands are degenerate at the Γ point for the case of an in-plane ferromagnetic monolayer CrI_3 [14]. We have obtained an indirect bandgap of 2.64 eV at the G_0W_0 level in the in-plane polarized structure, which is only slightly smaller than the direct bandgap at the Γ point (2.69 eV). The rotated magnetization has a strong impact on the polar MO signals, which can be understood from the symmetry. The dielectric function tensor $\boldsymbol{\varepsilon}$ is an axial tensor, which is in analogue to a dyad of two vectors. The broken C_3 rotational symmetry in an in-plane polarized structure leads to diminished amplitudes of ε_{xy} and ε_{yx}, because the x- and y-components are no longer correlated. We therefore expect small polar MO signals in an in-plane polarized structure. Our first-principles GW-BSE calculations have confirmed the above analysis as shown in Supplementary Figure 7f regarding to the polar MO Faraday effect. Moreover, we find that there are still strong excitonic effects in the in-plane case. In fact, we could still identify the three excitonic peaks (A, B and C) below the quasi-particle bandgap (Supplementary Figure 7d), with the exciton A having a binding energy of 1.29 eV, even larger than that in the out-of-plane case. This is probably because the high band degeneracy in the in-plane case increases the joint density-of-states around the Γ point, further enhancing the excitonic effects.

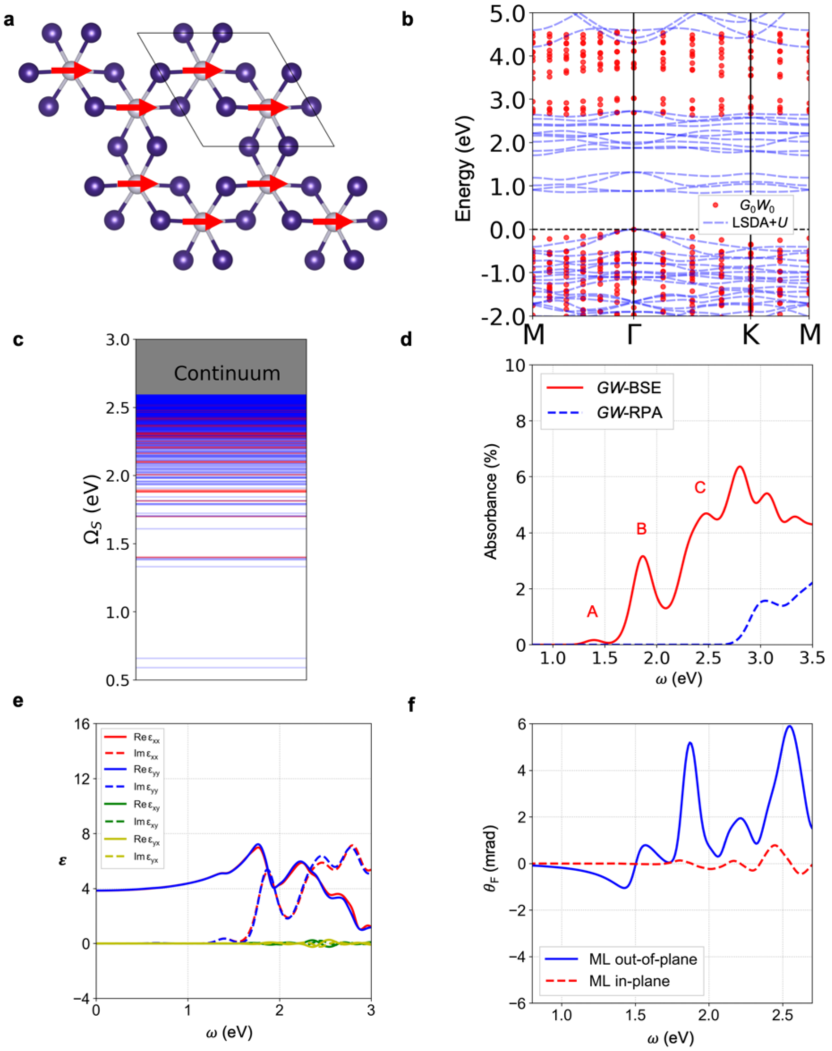

Supplementary Figure 7 | (**a**) Crystal structure and magnetization direction (red arrows) of an in-plane ferromagnetic monolayer CrI_3. (**b**) G_0W_0 (red solid) and LSDA+U (blue dashed) band structure of the in-plane ferromagnetic monolayer CrI_3, where a Hubbard onsite potential with U = 1.5 eV & J = 0.5 eV is adopted. (**c**) Exciton levels of in-plane ferromagnetic monolayer CrI_3 from GW-BSE calculations. Bright excitons are colored in red while dark ones in blue. The continuum starts from 2.69 eV. (**d**) Linearly polarized absorption spectrum at normal incidence with (GW-BSE, solid red) and without (GW-RPA, dashed blue) electron-hole interactions. An 80 meV energy broadening is adopted in (**d**) and the following plots in (**e**) and (**f**). (**e**) Calculated real part (solid lines) and imaginary part (dashed lines) of ε_{xx} (red), ε_{yy} (green), ε_{xy} (blue) and ε_{yx} (yellow) dielectric functions of in-plane ferromagnetic monolayer CrI_3. (**f**) Comparison between Faraday angle θ_F of an out-of-plane ferromagnetic monolayer CrI_3 and an in-plane ferromagnetic monolayer CrI_3 with the same P-FE configuration as in Fig. 4e in the main text.

K. Optical and MO properties of ferromagnetic bulk CrI_3

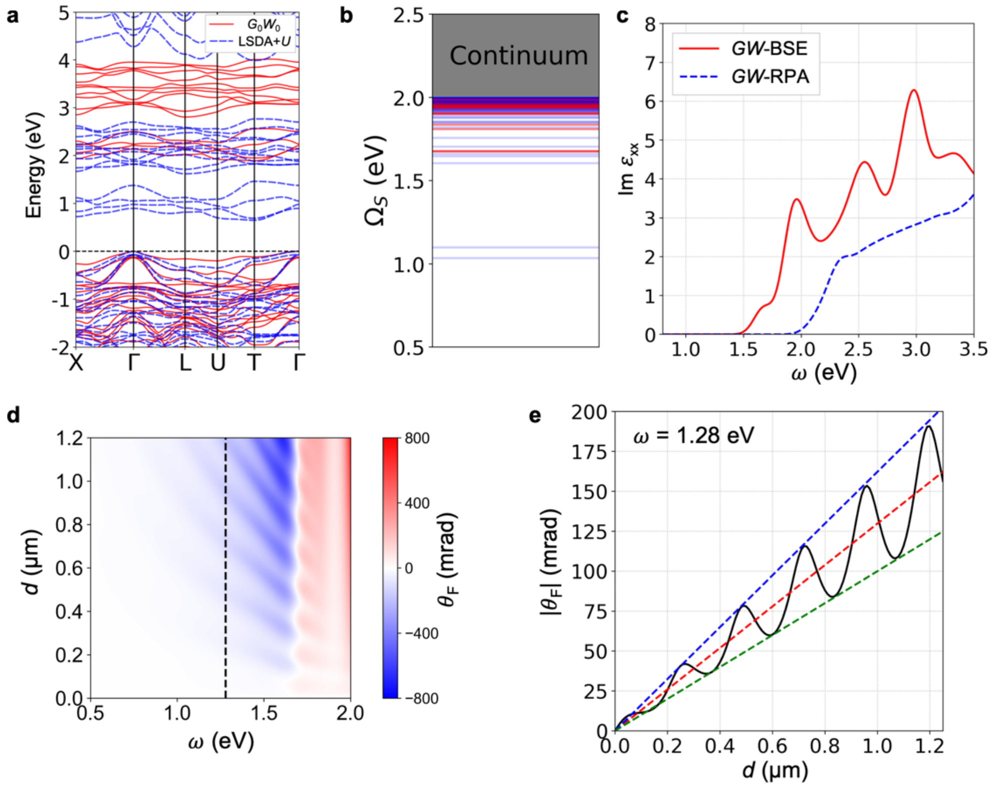

Supplementary Figure 8 | (a) G_0W_0 (red solid) and LSDA+U (blue dashed) band structure of ferromagnetic bulk CrI_3, where a Hubbard onsite potential with U = 1.5 eV and J = 0.5 eV is adopted. Each layer of bulk CrI_3 has the same out-of-plane direction as in monolayer CrI_3. The magnetization is along the out-of-plane direction. (**b**) Exciton levels of ferromagnetic bulk CrI_3 from *GW*-BSE calculations. Bright excitons are colored in red while dark ones in blue. The continuum starts from 2.0 eV. (**c**) Imaginary part of dielectric function ε_{xx} of ferromagnetic bulk CrI_3 with (solid red) and without (dashed blue) electron-hole interactions. An 80 meV energy broadening is adopted in (**c**) and following plots. (**d**) Thickness and frequency dependence of Faraday angle θ_{F} for a vacuum-CrI_3-vacuum device, where d refers to the thickness of the CrI_3 layer. (**e**) Thickness dependence of the amplitude of θ_{F} at $\omega = 1.28$ eV excitation, and the dashed lines are linear fits with slope: (blue) 1.6×10^3, (red) 1.3×10^3 and (green) 1.0×10^4 mrad·cm^{-1}.

L. Crystal structure and structure relaxation

We use the experimental structure for both ferromagnetic bulk and monolayer CrI_3. Bulk CrI_3 belongs to the space group R $\bar{3}$ (148) [15]. Both bulk and monolayer CrI_3 belong to the point group S_6. In fact, we have

checked the validity of the employed pseudopotentials with first-principles structure relaxation. During the relaxation, we used a kinetic energy cutoff of 120 Ry. The van der Waals interaction is included in two ways: the rVV10 nonlocal density functional [16] and the semiempirical Grimme's DFT-D3 method [17], both of which have been implemented in the Quantum ESPRESSO package [18]. The structures have been fully relaxed until the force on each atom is less than 0.02 eV/Å. The spin-orbit coupling effects are not considered in the relaxation. We find that there is little deviation between the relaxed bulk structure and the experimental bulk structure. In addition, the lattice constants and internal coordinates barely change from bulk to monolayer structures, indicating much stronger intralayer bondings than interlayer bondings. For these reasons, we use the experimental structure for both bulk and monolayer calculations. The detailed structure parameters are listed in Supplementary Table 3.

	Parameters (Å)	*a*	*c*	Cr-I distance	Interlayer distance	Intralayer thickness
**Bulk**	Exp. [30]	6.87	19.81	2.73	3.47	3.13
	rVV10	6.99	19.70	2.78	3.38	3.19
	DFT-D3	6.96	20.07	2.76	3.53	3.16
**Monolayer**	rVV10	6.98	N/A	2.78	N/A	3.21
	DFT-D3	6.96	N/A	2.76	N/A	3.18

Supplementary Table 3 | Structure parameters for ferromagnetic bulk and monolayer CrI_3. The lattice constants *a* and *c* refer to the conventional hexagonal cell.

The Quantum ESPRESSO structure input for bulk CrI_3 (with fractional coordinates) is listed below:

```
CELL_PARAMETERS angstrom
 3.9648952386328364   0.000000000000000  6.602333333333333
-1.9824476193164182   3.433700000000000  6.602333333333333
-1.9824476193164182  -3.433700000000000  6.602333333333333
ATOMIC_POSITIONS crystal
Cr   0.666330330  0.666330330  0.666330330
Cr   0.333669670  0.333669670  0.333669670
I   0.729082000  0.430059000  0.077768000
I   0.270918000  0.569941000  0.922232000
```

I 0.077768000 0.729082000 0.430059000

I 0.922232000 0.270918000 0.569941000

I 0.430059000 0.077768000 0.729082000

I 0.569941000 0.922232000 0.270918000

The Quantum ESPRESSO structure input for monolayer CrI_3 (with fractional coordinates) is listed below:

```
CELL_PARAMETERS angstrom
   6.8670000000    0.0000000000    0.0000000000
  -3.4335000000    5.9469964478    0.0000000000
   0.0000000000    0.0000000000   18.0000000000
ATOMIC_POSITIONS crystal
Cr  0.333333333  0.666666667  0.000377778
Cr  0.666666667  0.333333333  0.999622226
I  0.349896669  0.998803318  0.913106084
I  0.001196661  0.351093352  0.913106084
I  0.648906648  0.650103331  0.913106084
I  0.650103331  0.001196663  0.086893886
I  0.998803318  0.648906648  0.086893886
I  0.351093352  0.349896699  0.086893886
```

M. Dielectric function for quasi-2D materials

As an extensive physical quantity, the dielectric function is ill-defined for quasi-2D materials. In this work, we rescale the calculated dielectric function in a slab model by the thickness of a monolayer CrI_3 (d=c_{bulk}/3=6.6 Å),

$$\varepsilon_{xx} = 1 + \frac{l}{d}(\tilde{\varepsilon}_{xx} - 1), \tag{15}$$

and

$$\varepsilon_{xy} = \frac{l}{d}\tilde{\varepsilon}_{xy}, \tag{16}$$

where $\tilde{\varepsilon}_{xx}$ and $\tilde{\varepsilon}_{xy}$ are calculated dielectric functions from the slab model (monolayer CrI_3 with vacuum) with thickness l along the out-of-plane direction, and ε_{xx} and ε_{xy} are rescaled dielectric function for monolayer CrI_3 with monolayer thickness d.

To check the validity of our slab model calculations, we have performed calculations with different unit cell thickness l, and checked the scaling of the dielectric functions. As expected, we find a linear dependence of $(\varepsilon_{xx} - 1)$ and ε_{xy} on l, which justifies our rescaling scheme. Also, it should be noted that, according to Eqn. (5-14) the calculated MO signals do not depend on the choice of d, because the final expression of MO signals depends only on $d\varepsilon_{xy}$, which is invariant with different choice of d. We have checked the validity of our slab model calculations in Supplementary Figure 9 and Supplementary Figure 10.

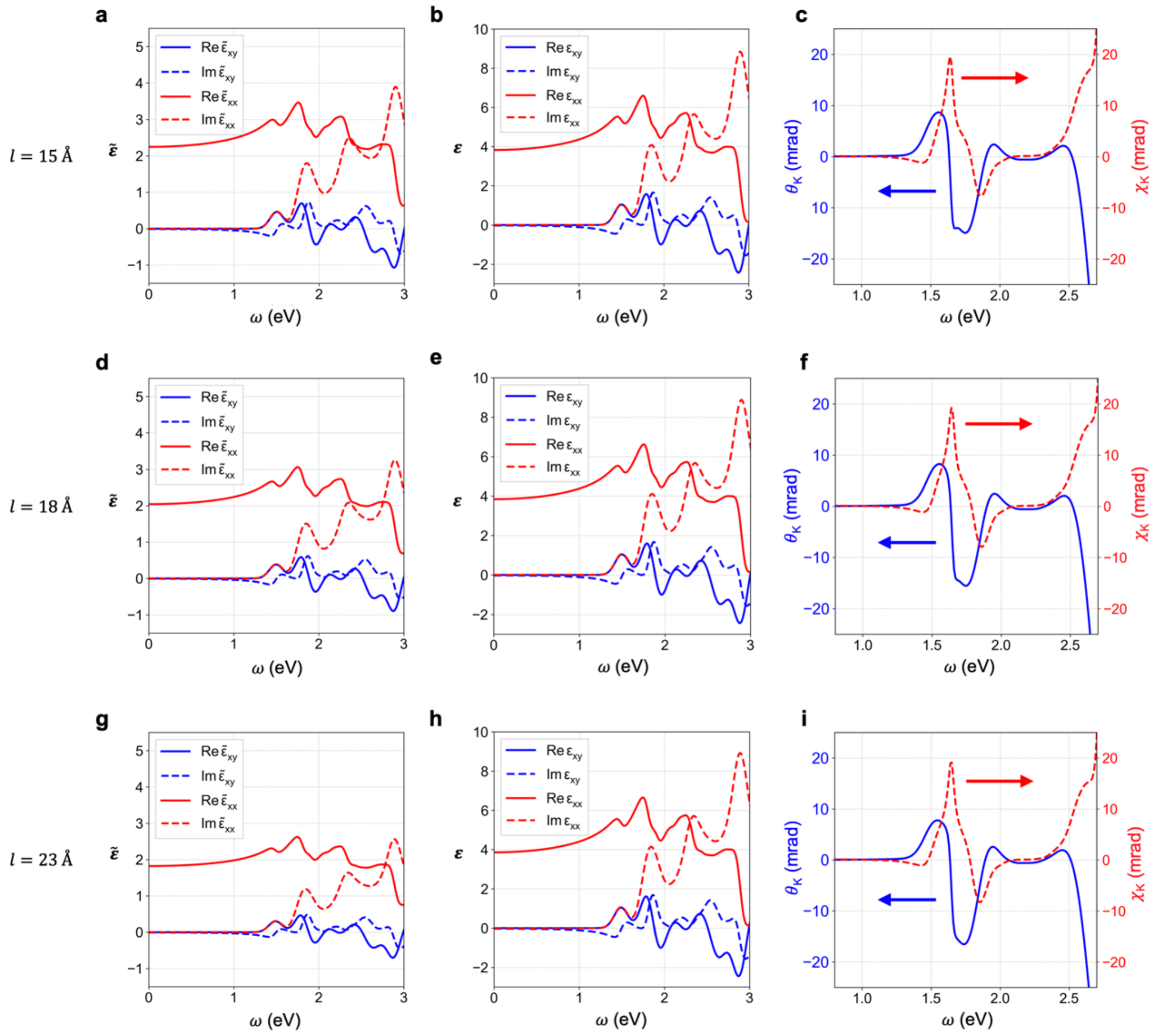

Supplementary Figure 9 | Calculated dielectric functions $\tilde{\varepsilon}$ of ferromagnetic monolayer CrI_3 in a slab model at *GW*-BSE level for (**a**) $l = 15$ Å, (**d**) $l = 18$ Å and (**g**) $l = 23$ Å. Rescaled dielectric function ε with $d = 6.6$ Å for (**b**) $l = 15$ Å, (**e**) $l = 18$ Å and (**h**) $l = 23$ Å. Kerr angles θ_{K} (left, solid blue curve) and Kerr ellipticity χ_{K} (right, dashed red curve) for the P-MOKE setup of vacuum-CrI_3-SiO_2(285 nm)-Si for (**c**) $l = 15$ Å, (**f**) $l = 18$ Å and (**i**) $l = 23$ Å. An 80 meV energy broadening is adopted.

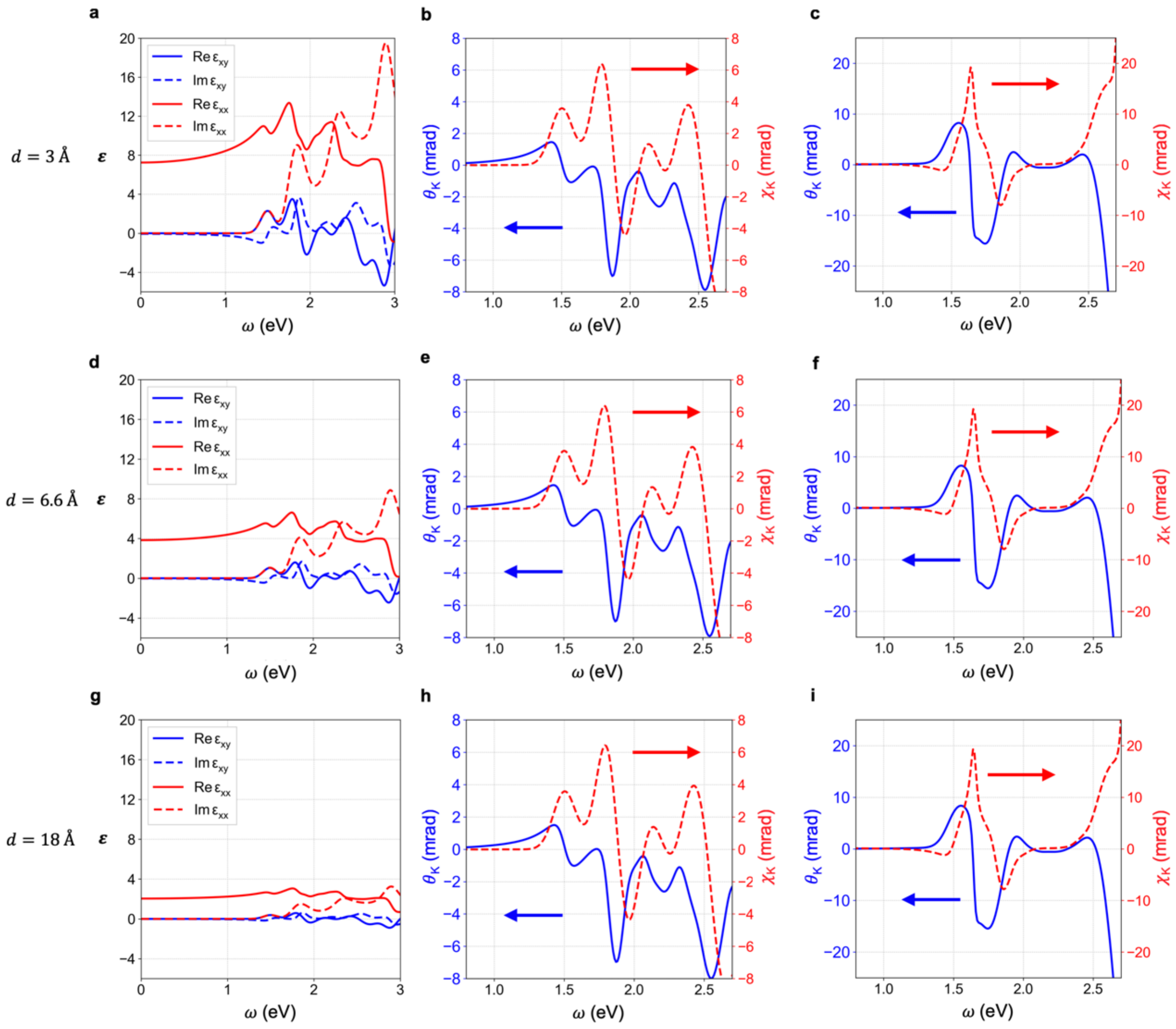

Supplementary Figure 10 | Rescaled dielectric functions ε of ferromagnetic monolayer CrI_3 in a slab model with $l = 18$ Å at *GW*-BSE level for (**a**) $d = 3$ Å, (**d**) $d = 6.6$ Å and (**g**) $d = 18$ Å. Kerr angles θ_K (left, solid blue curve) and Kerr ellipticity χ_K (right, dashed red curve) for the P-MOKE setup of vacuum-CrI_3-SiO_2 for (**b**) $d = 3$ Å, (**e**) $d = 6.6$ Å and (**h**) $d = 18$ Å. Kerr angles θ_K (left, solid blue curve) and Kerr ellipticity χ_K (right, dashed red curve) for the P-MOKE setup of vacuum-CrI_3-SiO_2(285 nm)-Si for (**c**) $d = 3$ Å, (**f**) $d = 6.6$ Å and (**i**) $d = 18$ Å. An 80 meV energy broadening is applied.

N. Effect of a hexagonal boron nitride (hBN) substrate on exciton energies

To study the effects of an insulating substrate, we perform *GW*-BSE calculations of a ferromagnetic monolayer CrI_3 on top of a monolayer hBN with an interlayer distance of 3.42 Å. The interlayer distance was determined with the van der Waals interaction through the DFT-D3 method [17]. It is expected that the substrate-induced exciton excitation energy redshift, albeit small, will quickly saturate with increasing thickness and additional layers of substrate will introduce negligible deviations [19-21]. Since hBN has a dielectric constant very similar to that of SiO_2 (both around 4), hBN and fused silica substrates of the same thickness should have similar screening effects on the exciton energies, as confirmed in previous work on transition metal dichalcogenides [19], as shown in Supplementary Figure 11.

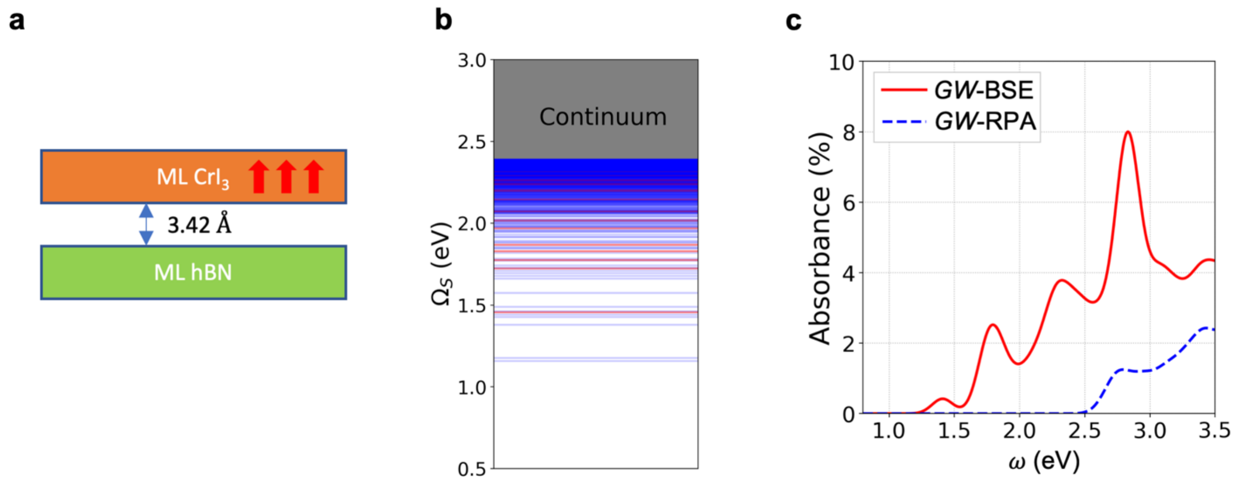

Supplementary Figure 11 | (**a**) Schematic of a ferromagnetic monolayer CrI_3 on top of a monolayer hexagonal BN substrate, with an interlayer distance of 3.42 Å. A Hubbard onsite potential with $U = 1.5$ eV & $J = 0.5$ eV is adopted. (**b**) Exciton levels of ferromagnetic monolayer CrI_3 with a monolayer hBN substrate from *GW*-BSE calculations. Bright excitons are colored in red while dark ones in blue. The continuum starts from 2.41 eV. (**c**) Linearly polarized absorption spectrum at normal incidence with (*GW*-BSE, solid red) and without (*GW*-RPA, dashed blue) electron-hole interactions. An 80 meV energy broadening is adopted.

O. Effects of *U* on single-particle energies

There is an interesting behavior of LSDA+*U* band energies with increasing *U*. As shown in the PDOS plots in Supplementary Figure 12, in contrast to the common impression of effects of +*U*, the bandgap

and the first conduction peak (both of which contain substantial I *p* orbital character) slightly decrease upon increasing *U*. Other Cr *d* states, on the other hand, show expected behaviors by going away from the Fermi level with increasing *U*.

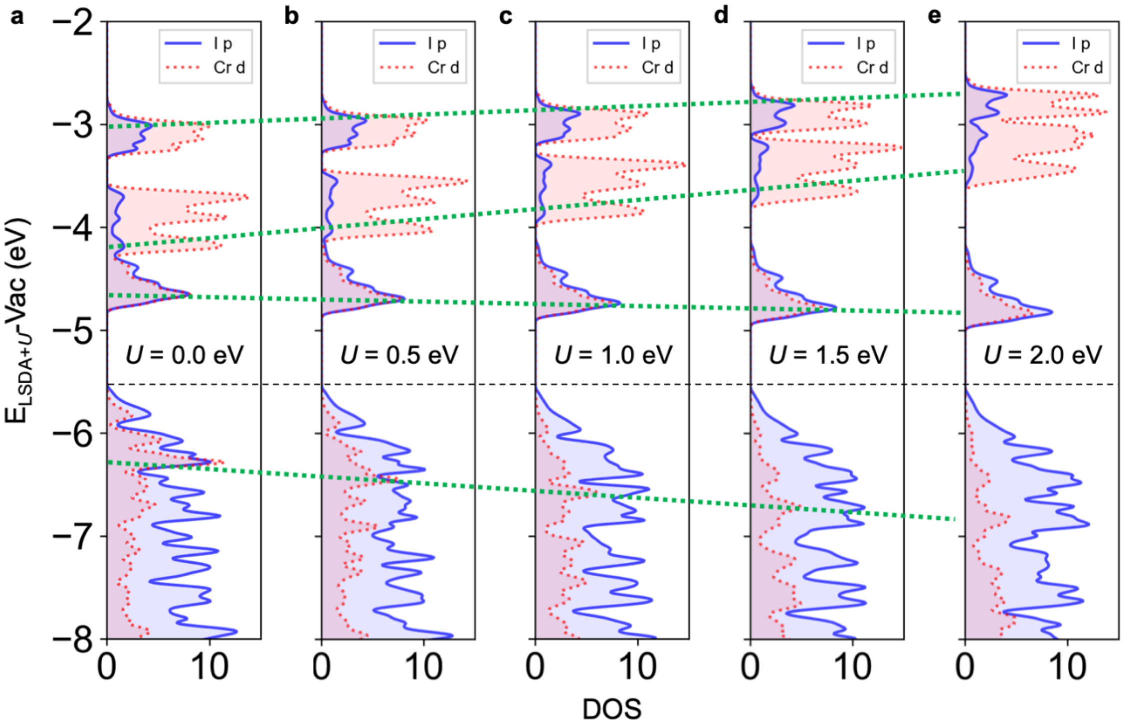

Supplementary Figure 12 | PDOS plots of ferromagnetic monolayer CrI_3 at the LSDA+*U* level with different *U* values from (a) *U* = 0 eV to (e) *U* = 2.0 eV. *J* is set to be zero in all cases for this illustrative investigation. The DOS is projected into contributions from Cr *d* (red) and I *p* (blue) orbitals. The green dots show the trends of band energies with increasing *U*. The single-particle energies have been aligned with the vacuum level in all the plots. The dashed black line indicates the VBM energy.

The effects of *U* could be understood in terms of the *Janak theorem*: the eigenvalue is the derivative of the total energy with respect to the occupation of a state i, $\epsilon_i = \frac{\partial E_{\text{tot}}}{\partial n_i}$, where i also includes the spin index [22]. In the case of LSDA+*U*, the total energy is given by $E_{\text{tot}} = E_{\text{LSDA}} + E_U - E_{\text{dc}}$ [1], and therefore the Bloch state eigenvalue can be expressed as,

$$\epsilon_{n\mathbf{k}} = \frac{\partial E_{\text{LSDA}}}{\partial n_{n\mathbf{k}}} + \frac{\partial (E_U - E_{\text{dc}})}{\partial n_{n\mathbf{k}}}. \tag{17}$$

To simplify our following discussions, we now assume *J* = 0. The energy correction from +*U* can be estimated as,

$$\Delta\epsilon_{n\mathbf{k}}^{+U} = \sum_{at,m\sigma} U\left(\frac{1}{2} - n_{m\sigma}\right) |\langle m\sigma|\psi_{n\mathbf{k}}\rangle|^2, \quad (18)$$

where $n_{m\sigma}$ is the occupation number for the *diagonalized* and spin-polarized local basis $|m\sigma\rangle$, and the Bloch states $|\psi_{n\mathbf{k}}\rangle$ are assumed to be fixed before and after $+U$. To get $n_{m\sigma}$ and $|m\sigma\rangle$, we first use atomic wave functions (five spin-up *d* orbitals and five spin-down *d* orbitals for each Cr atom) as projectors to build the local orbital-resolved spin density $n_{ll'}^{\sigma}$ by summing over all the contributions from occupied Bloch states, with the quantization axis chosen along the *z*-axis. Here $l, l' = 1, \dots, 5$ labels the *d* orbitals and $\sigma = \uparrow, \downarrow$ labels the spin polarization. We then diagonalize this spin density and get the eigenvalues n_m^{σ} and eigenvectors $|m\sigma\rangle$ as a linear combination of the five *d* orbitals with spin polarization σ. In this way, the eigenvalue correction should incorporate an extra factor to quantify how much the projection of the Bloch state is onto the localized basis. This is in contrast to the atomic limit, $\Delta\epsilon_i^{+U} = U(\frac{1}{2} - n_i)$, which indicates that the occupied and unoccupied states at the LSDA level are further split by U. But there is strong *p*-*d* hybridization in the CrI_3 systems, especially for e_g states which form σ bonds between Cr *d* orbitals and I *p* orbitals. In this way, the major-spin e_g states become delocalized, which means the overlap between the first set of conduction Bloch states and the localized atomic orbital $|m\sigma\rangle$ used in LSDA+U is small. To be explicit, $\Delta\epsilon_{n\mathbf{k}}^{+U}$ should be smaller for the e_g states than for the t_{2g} states, which has also been verified in our calculations. Moreover, the strong *p*-*d* hybridization makes the local occupation $n_{m\sigma}$ deviate from the atomic limit: that is, 3 major-spin *d* orbitals are completely occupied, while all the remaining *d* orbitals are empty. Here we used $U = 2.0$ eV in a case study. As listed in Supplementary Table 4, the spin density has a more uniform distribution than that in the atomic limit.

$U = 2$ eV & $J = 0$ eV	Minor spin					Major spin					$\Delta\epsilon_{+U}$ (eV)	
$n_{m\sigma}$	0.118	0.121	0.129	0.397	0.400	0.708	0.713	0.993	0.993	0.993	Janak	LSDA+U
$\lvert\langle n_{m\sigma} \mid v_1@\Gamma\rangle\rvert^2$	1.1E-5	2.4E-5	8.3E-5	1.1E-5	1.3E-4	1.6E-9	3.0E-5	6.1E-3	1.6E-5	1.2E-2	-0.04	-0.01
$\lvert\langle n_{m\sigma} \mid c_1@\Gamma\rangle\rvert^2$	7.4E-5	2.0E-6	6.6E-4	1.5E-6	1.2E-4	3.1E-7	2.2E-4	6.7E-2	1.6E-4	8.6E-2	-0.30	-0.16
$\lvert\langle n_{m\sigma} \mid c_5@\Gamma\rangle\rvert^2$	1.6E-1	1.6E-1	5.4E-2	6.5E-3	2.4E-2	6.4E-4	1.0E-5	1.0E-5	1.2E-6	1.2E-6	0.58	0.63
$\lvert\langle n_{m\sigma} \mid c_{14}@\Gamma\rangle\rvert^2$	1.9E-2	7.0E-3	3.6E-2	3.2E-6	2.0E-1	5.9E-6	2.4E-6	2.1E-5	9.1E-7	2.1E-5	0.17	0.27

Supplementary Table 4 | Occupation of diagonal basis on each Cr atom of a ferromagnetic monolayer CrI_3 from LSDA+U calculations with $U = 2.0$ eV & $J = 0$ eV. The local orbital-resolved spin density matrix $n_{ll'}^{\sigma}$ is diagonalized with eigenvalues $n_{m\sigma}$ as shown in the second row. The projections of the 1st

valence state and the 1st, 5th, 14th conduction states at the Γ point to these localized diagonal orbitals are calculated in the following rows. The Janak analysis and the LSDA+*U* results on the change in the band energy due to +*U* are listed in the last two columns.

Also note that the local spin density is completely determined from the occupied Bloch states, while the overlap between unoccupied Bloch states and occupied diagonal local basis ($n_{l\sigma} > 0.5$) is not necessarily zero. Indeed, our numerical results agree with our intuition that the e_g states will be more delocalized and have smaller overlap with localized diagonal orbitals. Also, the empty major-spin e_g states have small but noticeable overlap between the occupied major-spin diagonal orbitals, giving rise to the negative shift of CBM energy with increasing *U*, as confirmed from both our Janak analysis and DFT calculations (last two columns in Supplementary Table 4). Moreover, the p-dominant VBM state (v_1) at the Γ point has little overlap with those d-like localized diagonal orbitals, leading to negligible energy shift upon +*U*. For these reasons, the bandgap slightly decreases with increasing *U*.

Supplementary References

1. Liechtenstein, A. I. et al. Density-functional theory and strong interactions: Orbital ordering in Mott-Hubbard insulators. *Phys. Rev. B* **52**, R5467-R5470 (1995).
2. Miyake, T. et al. Quasiparticle energy of semicore *d* electrons in ZnS: combined LDA+*U* and *GW* approach. *Phys. Rev. B* **74**, 245213 (2006).
3. Kioupakis, E. et al. *GW* quasiparticle corrections to the LDA+*U* / GGA+*U* electronic structure of bcc hydrogen. *Phys. Rev. B* **77**, 155114 (2008).
4. Jiang, H. et al. First-principles modeling of localized *d* states with the *GW*@LDA+*U* approach. *Phys. Rev. B* **82**, 045108 (2010).
5. Mostofi, A. A. et al. Wannier90: A tool for obtaining maximally-localised Wannier functions. *Comput. Phys. Commun.* **178**, 685-699 (2008).
6. Deslippe, J. et al. BerkeleyGW: A massively parallel computer package for the calculation of the quasiparticle and optical properties of materials and nanostructures. *Comput. Phys. Commun.* **183**, 1269-1289 (2012).
7. Dunlap, W. C. Jr., & Watters, R. L. Direct measurement of the dielectric constants of silicon and germanium. *Phys. Rev.* **92**, 1396-1397 (1953).

8. Li, H. H. Refractive index of silicon and germanium and its wavelength and temperature derivatives. *J. Phys. Chem. Ref. Data* **9**, 561-658 (1980).
9. Gray, P. R. et al. Analysis and design of analog integrated circuits. Wiley (2001).
10. Oppeneer, P. M. Magneto-optical Kerr spectra. *Handbook of Magnetic Materials* **13**, 229-422 (2001).
11. Collett, E. Field guide to polarization. Vol. 15. Bellingham: SPIE press (2005).
12. Hecht, E. Optics. Chap. 9 Addison-Wesley (2001).
13. Byrnes, S. J. Multilayer optical calculations. Preprint at http://arxiv.org/abs/1603.02720.
14. Jiang, P. et al. Spin direction-controlled electronic band structure in two-dimensional ferromagnetic CrI_3. *Nano Lett.* **18**, 3844-3849 (2018).
15. McGuire, M. A. et al. Coupling of crystal structure and magnetism in the layered, ferromagnetic insulator CrI_3. *Chem. Mater.* **27**, 612-620 (2015).
16. Sabatini, R. et al. Nonlocal van der Waals density functional made simple and efficient, *Phys .Rev. B* **87**, 041108(R) (2013).
17. Grimme, S. et al. A consistent and accurate *ab initio* parametrization of density functional dispersion correction (DFT-D) for the 94 elements H-Pu, *J. Chem. Phys.* **132**, 154104 (2010).
18. Giannozzi, P. et al. QUANTUM ESPRESSO: A modular and open-source software project for quantum simulations of materials. *J. Phys. Condens. Matter* **21**, 395502 (2009).
19. Raja, A. et al. Coulomb engineering of the bandgap and excitons in two-dimensional materials. *Nat. Commun.* **8**, 15251 (2017).
20. Ugeda, M. M. et al. Giant bandgap renormalization and excitonic effects in a monolayer transition metal dichalcogenide semiconductor. *Nat. Mater.* **13**, 1091-1095 (2014).
21. Qiu, D. Y. et al. Environmental screening effects in 2D materials: renormalization of the bandgap, electronic structure and optical spectra of few-layer black phosphorus. *Nano Lett.* **17**, 4706-4712 (2017).
22. Janak, J. F. Proof that $\frac{\partial E}{\partial n_i} = \epsilon$ in density-functional theory. *Phys. Rev. B* **18**, 7165-7168 (1978).
